# TRPV1 Induced Apoptosis of Colorectal Cancer Cells by Activating Calcineurin-NFAT2-p53 Signaling Pathway

**DOI:** 10.1155/2019/6712536

**Published:** 2019-04-30

**Authors:** Nengyi Hou, Xuelai He, Yuhui Yang, Junwen Fu, Wei Zhang, Zhiyi Guo, Yang Hu, Liqin Liang, Wei Xie, Haibo Xiong, Kang Wang, Minghui Pang

**Affiliations:** ^1^Department of Gastrointestinal Surgery, Sichuan Provincial People's Hospital, University of Electronic Science and Technology of China, Chengdu, China; ^2^Department of General Surgery, No. 4 Hospital, Zigong City, Sichuan Province, China

## Abstract

**Background/Aims:**

TRPV1 is a nonselective Ca^2+^ channel which has recently been observed in many cancers, while its effect on cell proliferation, apoptosis, metabolism, and cancer development in colorectal cancer (CRC) is still unclear. In this study, we hypothesized that TRPV1 is a tumor suppressor in CRC development as well as the underlying mechanism.

**Methods:**

Immunohistochemistry assay was applied to detect the expression of TRPV1 protein in CRC tissues. HCT116 cell proliferation and apoptosis were measured by CCK-8 and flow cytometry, respectively. Cellular Ca^2+^ concentration was measured by Fluo-4/AM-based flow cytometer. Apoptosis-related proteins were measured by Western blotting.

**Results:**

In this study, we found that TRPV1 expression was significantly decreased in CRC tissues, compared with CRC-adjacent tissues and normal tissues, respectively. Then, we found that the TRVP1 agonist capsaicin treatment inhibited CRC growth and induced apoptosis by activating P53. Subsequent mechanistic study revealed that the TRPV1 induced cytosolic Ca^2+^ influx to regulate cell apoptosis and p53 activation through calcineurin.

**Conclusions:**

This study suggests that TRPV1 served as a tumor suppressor in CRC and contributed to the development of novel therapy of CRC.

## 1. Introduction

Colorectal cancer (CRC) is one of the common malignant tumors of digestive system and its incidence is increasing year by year and has been reported as the fourth leading cause of cancer associated death worldwide [1]. Currently, research on the pathogenicity and the underlying molecular mechanism of colorectal cancer is still in its infancy [2]. The occurrence of colorectal cancer needs to undergo the process of normal mucosal epithelium to adenoma, dysplasia, carcinoma in situ, colorectal adenocarcinoma, and metastatic carcinoma [3], involved in multiple genes.

Therefore, in-depth research on the genetic and molecular mechanisms related to the occurrence and development of colorectal cancer is an important theoretical basis for the prevention and treatment of colorectal cancer in the future.

Transient receptor potential oxalic acid subtype 1 (TRPV1) is a nonselective cationic ligand gate channel, belonging to the family of transient receptor potential (TRP) ion channels, as it can be activated by capsaicin, also known as vanilloid receptor 1 [4]. TRPV1 was initially regarded as a key sensor for responses to heat and mechanical and chemical stimuli due to its dominance in afferent sensory neurons [5]. TRPV1, meanwhile, has also been linked to the metabolism, longevity, inflammation, and cancer [6]. Yang Y et al. reported that the low expression of TRPV1 was contributed to melanoma growth via calcineurin-ATF3-p53 pathway [7]. Conversely, the TRPV1 was highly expressed in prostatic cancer, and the lack of TRPV1 inhibited the spread of prostate cancer cells [8]. The above studies showed that the effect of TRPV1 is related to tumor type. Previous reports have shown that TRPV1 is closely related to intestinal diseases such as translocation, irritable bowel syndrome, and colitis [9, 10]. Recently, studies have shown that inhibiting TRPV1 can increase the apoptosis sensitivity of colorectal cancer cells by regulating the ROS-JNK-CHOP pathway [11]. However, the mechanism of TRPV1 inducing apoptosis of colorectal cancer cells remains to be further studied.

Ca^2+^ is among the major second messengers for connecting membrane receptor activation and downstream signaling transduction, playing an important role in many fundamental physiological processes, including cell excitability, vitality, apoptosis, and transcription [12]. Recent studies have shown that Ca^2+^ also contributes to some malignant behaviors in tumors, such as proliferation, invasion, migration, and metastasis [13]. The imbalance of intracellular Ca^2+^ influx is closely related to the hallmarks of various cancers including colorectal cancer [14, 15]. Given that TRPV1 is a powerful nonselective Ca^2+^ channel, we hypothesize that TRPV1 can affect the growth of colorectal cancer cells by regulating Ca^2+^ dependent signaling. Thus, the aim of the present study was to investigate the role of TRPV1 in colorectal cancer progression and provide a deeper understanding of the causal mechanisms of cancer cell proliferation and apoptosis.

## 2. Materials and Methods

### 2.1. Reagents

Capsaicin (8-methyl-N-vanillyl-trans-6-nonenamide), Pifithrin-*α* (2-(2-Imino-4, 5, 6, 7-tetrahydrobenzothiazol-3-yl)-1-p-tolylethanone hydrobromide), and FK506 monohydrate (Tacrolimus) were all obtained from Sigma-Aldrich (Merck KGaA, Darmstadt, Germany). Additional reagents employed in the present study were commercially available and of analytical purity.

### 2.2. Tissue Samples

A cohort of 10 colorectal cancer (CRC) tissue samples, 10 CRC-adjacent tissue samples, and 6 normal subjects for protein detection was obtained from Sichuan Provincial People's Hospital according to the institutional guidelines. All volunteers signed the informed consent. This study was approved by the Ethics Review Board at the University of Electronic Science and Technology of China (Chengdu, China).

### 2.3. Immunohistochemistry

Immunohistochemical analysis was performed on paraffin-embedded tissues sections. The antigen retrieval was performed by using 3% hydrogen peroxide at room temperature for 15 min. Subsequently, the sections were incubated with appropriate primary antibody TRPV1 (Cell Signaling Technology, MA, USA) at 4°C overnight. Following rewarmed for 30 min in a 37°C incubator, the sections were incubated with appropriate amount of biotinylated goat anti-rabbit IgG for 30 min at 37°C. The SABC-POD Kit (Beijing Solarbio Science & Technology Co., Ltd., Beijing, China) was used for immunohistochemistry of TRPV1 and then counterstained with hematoxylin. PBS was adopted to substitute for primary antibody as negative control group. A total of five visual fields (magnification, ×100 and ×400) in each section were randomly selected by using a Nikon computer image system (Nikon, Tokyo, Japan) and then assessed for immunoreactive areas using Image-Pro Plus software.

### 2.4. Cell Viability Assay

The human colorectal cancer cell line HCT116 was obtained from the Shanghai Institutes of Biological Sciences, Chinese Academy of Sciences (Shanghai, China). HCT116 cells were cultured in DMEM medium supplemented with 10% Fetal Bovine Serum (FBS; Gibco, CA, USA), incubated at 37°C in 5% CO_2_.

Cell viability was determined by Cell Counting Kit-8 (CCK-8; Dojindo Laboratories, Kumamoto, Japan) assay. In general, approximately 7x10^3^ cells per well were seeded in 96-well plates. Following incubation, the original culture medium was removed and 100 *μ*l fresh medium was mixed with CCK-8 at a ratio of 10:1 which was added to each well for 30 min at 37°C. The absorbance value was measured at 450 nm by a microplate reader (Bio-Rad, CA, USA).

### 2.5. Annexin V/Propidium Iodide (PI) Double-Staining Assay

An Annexin V-fluorescein isothiocyanate- (FITC-) PI Apoptosis Detection Kit (BD Pharmingen; BD Biosciences, Franklin Lakes, NJ, USA) was used to detect apoptosis. HCT116 cells were incubated with Capsicin, Pifithrin-*α*, or FK506 before collection for apoptosis detection. For detecting purpose, cells were collected and resuspended in 100 *μ*l binding buffer (1x10^5^cells) with 5 *μ*l Annexin V-FITC and 5 *μ*l PI (BD Biosciences, Franklin Lakes, CA, USA) and incubated at room temperature (20-25°C) for 15 min in the dark. Cell apoptosis was detected using a FACSCalibur™ Flow Cytometer (BD Biosciences) within 1 h.

### 2.6. Western Blotting Assay

Cells specimens were lysed using RIPA lysis buffer (Boster, Wuhan, China). The equal amounts of total proteins were separated by 10% SDS-PAGE gel and then transferred onto a PVDF membrane (Millipore, MA, USA). The membranes were blocked with 5% skim milk powder at room temperature for 1 h and incubated with primary antibodies diluted in blocking buffer at 4°C overnight. Subsequently, the membranes were washed and incubated with the appropriate HRP-conjugated secondary antibodies for 1 h at room temperature. Protein bands were detected by an ECL chemiluminescence kit (Millipore) according to the manufacturer's instructions. Protein levels were calculated relative to *β*-actin. Primary antibodies were used as follows: rabbit anti-NFAT2 (phospho S237) (Abcam, Cambridge, UK, ab183023), rabbit anti-p53 (#2527), rabbit anti-Bax (#5023), rabbit anti-Bcl-2 (#4223), rabbit anti-cleaved-caspase-3 (#9664), rabbit anti-NFAT2 (#8032), and rabbit anti-*β*-actin (#4970) were all from cell signaling (Danvers, MA, USA).

### 2.7. Cellular Ca^*2*+^ Concentration Determination

To measure intracellular Ca^2+^ in colon cancer cells, we used Fluo-4/AM, a cell-permeable fluorescent Ca^2+^ indicator. Briefly, the HCT116 cells were seeded at a density of 3x10^4^/well in 12-well plates and treated with capsaicin for 24 h. The cells were washed with Hanks Balanced Salt Solutions (HBSS) three times and then stained with 2 *μ*M Fluo-4/AM for 30 min at 37°C. The results were then evaluated with a flow cytometer. Experiments were repeated at least three times.

### 2.8. Immunofluorescence Assay

HCT116 cells were plated in 12-well plates and incubated overnight for adherence. Subsequently, cells were fixed in 4% paraformaldehyde for 10 min at room temperature, permeabilized with 0.1% Triton X-100, blocked with 5% BSA, and incubated with primary rabbit anti-NFAT2 antibody (cell signaling, MA, USA) 1h at room temperature. Slides were washed twice with PBS/0.1% Tween20 and incubated with a secondary AlexaFluor 488-conjugated anti-rabbit (green color; Invitrogen, CA, USA) for 1h at room temperature. Analyses were performed using ImageJ Software (NIH, Bethesda, MD, USA).

### 2.9. Statistical Analysis

Statistical analysis was performed using SPSS20.0 software (IBM Corp., Armonk, NY, USA). All data are presented as the mean ± standard deviation. Differences among multiple groups were compared by one-way analysis of variance (ANOVA) with Dunnett's posttests or two-way ANOVA with Bonferroni's posttests, and differences between two groups were compared by the Dunnett-t test. P < 0.05 was considered statistically significant.

## 3. Results

### 3.1. The Expression of TRPV1 Is Decreased in CRC Tissues

**Figure 1:**
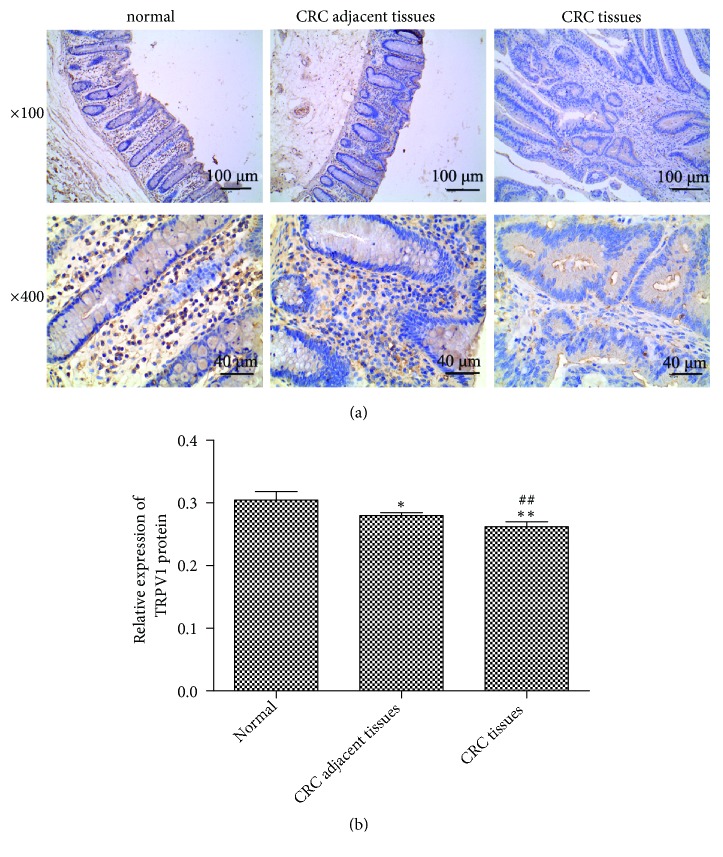
*Decrease of TRPV1 expression level in CRC*. (a) Immunohistochemical assay was performed to detect the expression of TRPV1 in CRC tissues and adjacent tissues. (b) The expression change of TRPV1 was statistically analyzed (CRC tissues_n=10_, CRC-adjacent tissues_n=10_, and normal tissues_n=10_). The result was shown as means ± standard deviation.  ^*∗*^p < 0.05 and  ^*∗∗*^p < 0.01 versus normal group. ^##^p < 0.01 versus CRC-adjacent tissues group.

To investigate whether TRPV1 is dysregulated in colorectal cancer, we detected the endogenous level of TRPV1 in CRC tissues by using Immunohistochemical assay. As shown in Figure 1, the protein level of TRPV1 was lower in CRC and adjacent tissues, compared with normal tissues. The protein level of TRPV1 in CRC tissues was significantly decreased compared with the adjacent tissues. These results demonstrated a significant decrease of TRPV1 expression in CRC, suggesting that TRPV1 may be a tumor suppressor.

### 3.2. TRPV1 Induced CRC Cell Proliferation Inhibition and Apoptosis by Activating p53

**Figure 2:**
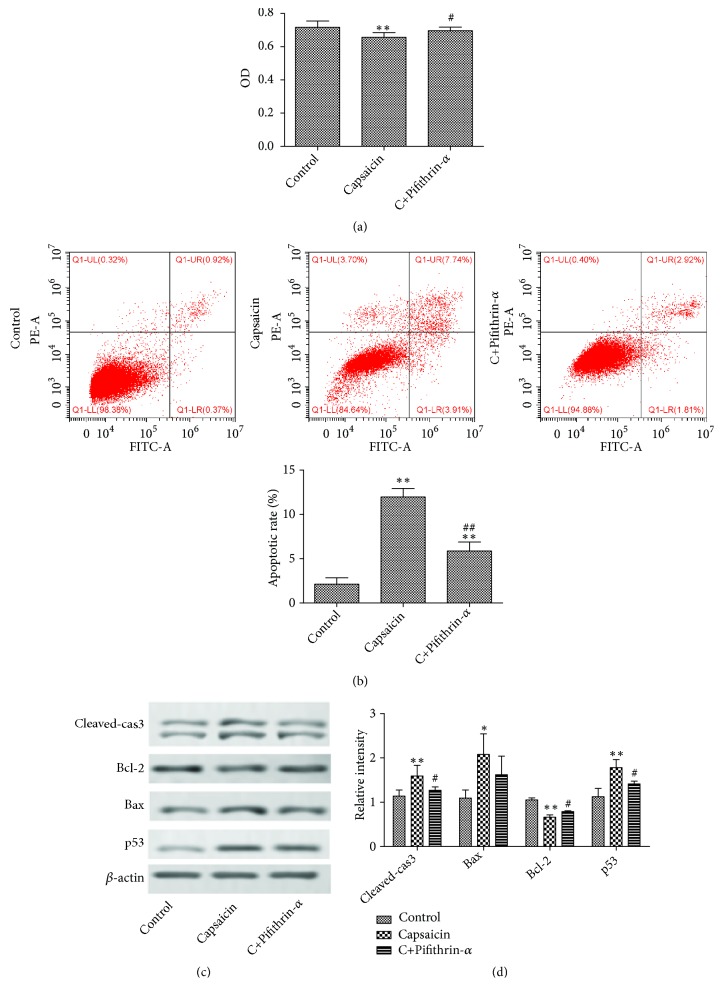
*TRPV1 promoted CRC cell apoptosis through activating p53*. HCT116 cells were incubated with capsaicin (50 *μ*M) in the absence or presence of Pifithrin-*α* (20 *μ*M). (a) Cell viability was determined by CCK-8 assay following indicated treatment. (b) Cell apoptosis were detected by flow cytometry. (c) The expression levels of apoptosis-related protein were examined by Western blotting. (d) The relative intensity was shown as a bar graph. The result was shown as means ± standard deviation.  ^*∗*^p < 0.05 and  ^*∗∗*^p < 0.01 versus control group. ^##^p < 0.01 and ^#^p < 0.05 versus capsaicin group.

To explore the role of TRPV1 in CRC growth, the CRC cell line HCT 116 was treated with capsaicin, a powerful TRPV1 agonist. As a result, capsaicin treatment significantly inhibited HCT116 cell proliferation and induced cell apoptosis, while the proliferation inhibition and apoptosis of HCT 116 cells were significantly decreased following pretreatment with Pifithrin-*α*, a powerful p53 inhibitor, compared with capsaicin group (Figures 2(a) and 2(b)). Furthermore, TRPV1 activation led to prominent upregulation of Bax, cleaved-caspase 3 and p53, and downregulation of Bcl-2, while the apoptosis-related protein expression was inhibited following pretreatment with Pifithrin-*α* (Figures 2(c) and 2(d)). These results indicated that the TRPV1 inhibited CRC cell proliferation and induced CRC cell apoptosis through activating p53.

### 3.3. TRPV1 Increased Cytosolic Ca^2+^ Influx and NFAT Protein Expression Level

**Figure 3:**
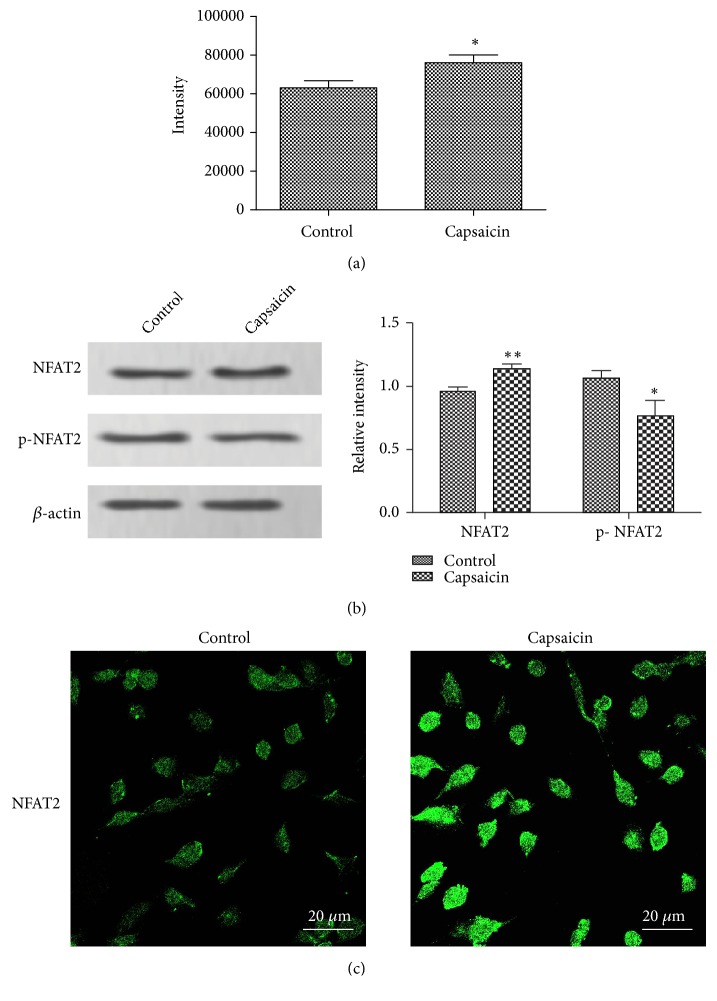
*Increased cytosolic Ca* ^*2+*^ *influx and NFAT protein induced by TRPV1 overexpression*. (a) Measurement of Ca^2+^ influx by staining with Fluo-4 AM in HCT116 cells following treated with capsaicin (50 *μ*M) and then detected by using flow cytometer. (b) The expression levels of p-NFAT2 and NFAT2 protein were examined by Western blotting, and the relative intensity was shown as a bar graph. (c) The expression level of NFAT2 protein was examined by immunofluorescence assay; images were observed by fluorescence microscopy (magnification, ×400). The result was shown as means ± standard deviation.  ^*∗*^p < 0.05 and  ^*∗∗*^p < 0.01.

We then investigated the mechanism of TRPV1 in regulating HCT116 cell apoptosis. First, we used Fluo-4/AM-based flow cytometer to measure intracellular calcium concentration following TRPV1 activation. As shown in Figure 3(a), the intensity of fluorescence was significantly increased following capsaicin treatment, indicating the upregulation of intracellular Ca^2+^ concentration. Western blotting analysis showed that p-NFAT2 was significantly downregulated following TRPV1 activation, with the NFAT2 protein expression level increased (Figure 3(b)). In parallel, the immunofluorescence analysis showed obvious increase of NFAT2 (Figure 3(c)).

### 3.4. TRPV1 Promoted CRC Cell Apoptosis by Activating Calcineurin

**Figure 4:**
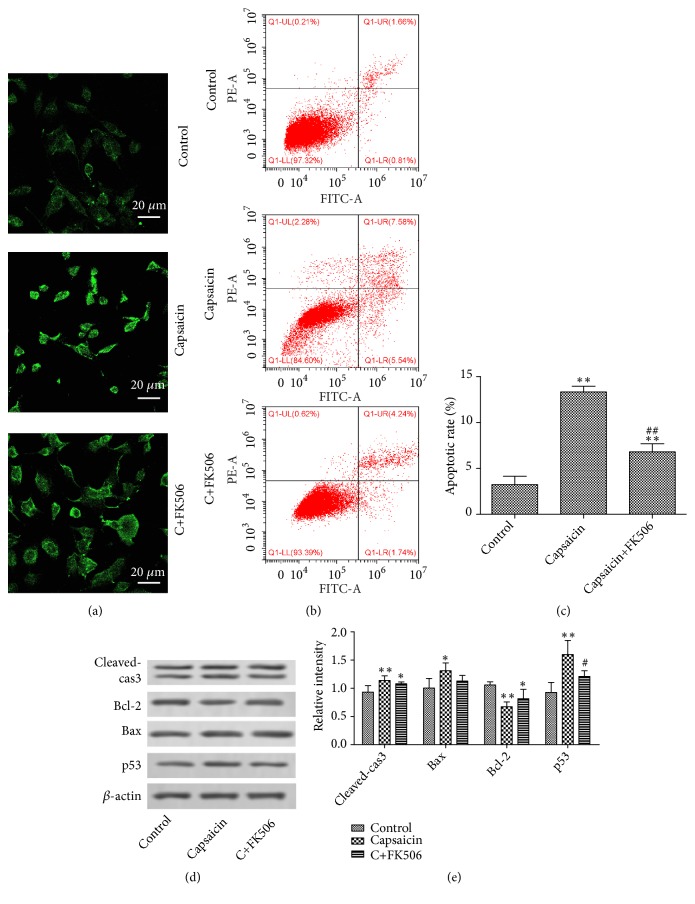
*TRPV1 induced cell apoptosis through activating calcineurin*. HCT116 cells were incubated with capsaicin (50 *μ*M) in the absence or presence of FK506 (4 *μ*M). (a) The expression level of NFAT2 protein was examined by immunofluorescence assay; images were observed by fluorescence microscopy (magnification, ×400). (b) Cell apoptosis were detected by flow cytometry. (c) The expression levels of apoptosis-related protein were examined by Western blotting. (d) The relative intensity was shown as a bar graph. The result was shown as means ± standard deviation.  ^*∗*^p < 0.05 and  ^*∗∗*^p < 0.01 versus control group. ^##^p < 0.01 and ^#^p < 0.05 versus capsaicin group.

We forwardly investigated whether TRPV1 regulated CRC cell apoptosis via calcineurin. The CRC cell line HCT116 was treated with FK506, an inhibitor of calcineurin, following capsaicin treatment. As shown in Figure 4(a), the treatment of FK506 led to decrease of NFAT2 protein compared with calcineurin group, indicating it was functional in suppressing calcineurin activation. Then, flow cytometer results showed that the FK506 significantly reversed the effect of capsaicin on cell apoptosis (Figures 4(b) and 4(c)). In addition, the apoptosis-related protein including Bax, cleaved-caspase 3 and p53 and antiapoptosis protein Bcl-2 was altered correspondingly (Figures 4(d) and 4(e)). These results suggested that TRPV1 promoted cell apoptosis and p53 activation through activating calcineurin.

## 4. Discussion

TRPV1 is a ligand-gated Ca^2+^-permeable ion channel and involved in Ca^2+^ transport and then maintains the introcellular calcium level [16]. The effects of TRPV1 expression on tumorigenesis and prognosis are different in various types of tumor. The decrease of TRPV1 expression in renal cell carcinoma was significantly associated with tumor Fuhrman grades and histopathological subtypes [17], while the intracellular aggregated TRPV1 was associated with lower survival in breast cancer patients [18]. At present study, we first found that the TRPV1 was lowly expressed in CRC tissues compared with CRC-adjacent tissues and normal subjects, which prompted us to speculate TRPV1 as a tumor suppressor. To clarify the role of TRPV1 in CRC, HCT116 cells were treated with capsaicin, a powerful TRPV1 agonist. As expected, capsaicin treatment markedly inhibited the proliferation of HCT116 cells. Apoptosis is a natural barrier against cancer. Our study showed that the capsaicin treatment significantly induced cell apoptosis and led to prominent upregulation of apoptosis-related proteins. Furthermore, to confirm whether TRPV1 promoted cell apoptosis via p53, we first inhibited p53 expression by Pifithrin-*α* and then treated with capsaicin. As a result, the proliferation inhibition and apoptosis of HCT116 cells were significantly decreased following pretreatment with Pifithrin-*α*, indicating that the TRPV1, the activation of P53, mediated the proapoptotic role of TRPV1 in CRC.

Ca^2+^ is known as an essential second messenger that controls various cell physiological functions. Since TRPV1 is a nonselective Ca^2+^ channel and intracellular Ca^2+^ homeostasis is critical for the survival and growth of colorectal cancer cells [19], we speculated that Ca^2+^-dependent effectors were involved in. At present study, we found that the intensity of fluorescence was significantly increased in HCT116 cells following capsaicin treatment, indicating the upregulation of intracellular Ca^2+^ concentration. Calcineurin is among the most canonical transponders of Ca^2+^-dependent signal transduction cascade involved in the control of cell cycle progression [20] and cell apoptosis [21] through the dephosphorylation and subsequent nuclear translocation of the downstream transcriptional factor NFAT2 [22]. The Ca^2+^/Calcineurin/NFAT signaling plays an important role in cancerogenesis [23] and served as a novel therapeutic target in leukemia and solid tumors [24]. Therefore, we wondered whether calcineurin mediated the effect of TRPV1. Our study showed that the phosphorylated NFAT2 was markedly downregulated following capsaicin treatment, with the NFAT2 protein expression level increased, indicating the activation of calcineurin. We forwardly investigated whether TRPV1 regulated CRC cell apoptosis via calcineurin. FK506, an inhibitor of calcineurin, acts via the complex of FK506 and FK506-binding protein binding to calcineurin to prevent calcineurin-mediated dephosphorylation, commonly used in treating various autoimmune diseases [25]. At present study, we found that the treatment of FK506 led to decrease of NFAT2 protein in HCT116 cells, indicating that it was functional in suppressing calcineurin activation. The flow cytometer results showed that the FK506 significantly reversed the effect of capsaicin on cell apoptosis, and the apoptosis-related proteins were altered correspondingly, indicating that the TRPV1 promoted cell apoptosis and p53 activation through activating calcineurin.

In summary, we measured the TRPV1 with low expression in CRC tissues and demonstrated that the overexpression of TRPV1 by capsaicin treatment could inhibit cell and increase cell apoptosis in HCT116 cells through activating p53. Moreover, the proapoptotic effect of TRPV1 was attributed to the increased Ca^2+^ influx and activation of calcineurin. Taken together, we demonstrated TRPV1 as a potent tumor suppressor by activating calcineurin-NFAT2-p53 signaling pathway, potentially offering new molecular targets for treatment of CRC.

## Data Availability

The datasets used or analyzed during the current study are available from the corresponding author on reasonable request.
